# Ion Channels of Nociception

**DOI:** 10.3390/ijms21103553

**Published:** 2020-05-18

**Authors:** Rashid Giniatullin

**Affiliations:** A.I. Virtanen Institute, University of Eastern Finland, 70211 Kuopio, Finland; Rashid.Giniatullin@uef.fi; Tel.: +358-403553665

**Keywords:** pain, nociception, sensory neurons, ion channels, P2X3, TRPV1, TRPA1, ASIC, Piezo channels, migraine, tooth pain

## Abstract

The special issue “Ion Channels of Nociception” contains 13 articles published by 73 authors from different countries united by the main focusing on the peripheral mechanisms of pain. The content covers the mechanisms of neuropathic, inflammatory, and dental pain as well as pain in migraine and diabetes, nociceptive roles of P2X3, ASIC, Piezo and TRP channels, pain control through GPCRs and pharmacological agents and non-pharmacological treatment with electroacupuncture.

Sensation of pain is one of the fundamental attributes of most species, including humans. Physiological (acute) pain protects our physical and mental health from harmful stimuli, whereas chronic and pathological pain are debilitating and contribute to the disease state.

Despite active studies for decades, molecular mechanisms of pain—especially of pathological pain—remain largely unaddressed, as evidenced by the growing number of patients with chronic forms of pain. There are, however, some very promising advances emerging. A new field of pain treatment via neuromodulation is quickly growing, as well as novel mechanistic explanations unleashing the efficiency of traditional techniques of Chinese medicine. New molecular actors with important roles in pain mechanisms are being characterized, such as the mechanosensitive Piezo ion channels [1]. 

Pain signals are detected by specialized sensory neurons, emitting nerve impulses encoding pain in response to noxious stimuli. Many of these nociceptive neurons are equipped with a rich repertoire of specific ion channels which serve as pain transducers. These ion channels are located at the peripheral terminals of dorsal root or trigeminal ganglia neurons as well as in sensory neurons of the viscera (Figure 1). Pain transducers react to a variety of chemical or physical stimuli (algogens) by opening the ion channels and inducing neuronal depolarization known as the generator potential. These pain transducers include ATP-gated P2X3, classical heat/capsaicin-sensitive TRPV1 and cold/redox-sensitive TRPA1 channels, acid sensitive ion channels (ASICs), and mechanosensitive Piezo, to name just a few (Figure 1, for further details see the classical review [2]). Sufficiently high generator potential, assisted by voltage-gated sodium channels, initiates the propagating action potentials (spikes). Some voltage-gated channels are expressed exclusively in nociceptive neurons, for instance, sodium Nav1.8 and Nav1.9 channels [3]. The peripheral pain signals travel via the multi-synaptic network of the nociceptive system to the higher pain centers to be perceived as a feeling of pain. 

A remarkable property of nociceptive neurons is sensitisation (enhanced responsiveness to triggers of pain). This phenomenon, a type of neuronal plasticity, can be mediated by a plethora of metabotropic receptors for different classes of pain modulators: classical mediators, neurotrophins, cytokines, and other active molecules (Figure 1). Normally, many pain transducers are in “sleeping” or low activity mode, but they can become very active during the action of these endogenous modulators triggering neuronal sensitization. 

The fundamental approach: “treat pain at the source” provides a strategic rationale to diminish pain by counteracting its peripheral mechanisms. The knowledge of the function and structure of pain transducers and associated ion channels is essential to develop new medicines. The fact that the molecular structures of most ion channels implicated in nociception are known facilitates the development of new anti-nociceptive (analgesic) medicines. Direct targeting of pain transducers by specific blockers provides an opportunity to block pain mediated by these ion channels. However, this is a challenging task since the lifetime of channels on the membrane is limited because of the continuous traffic and renewal as it was shown for the nociceptive P2X3 receptors [4].

This special issue “Ion Channels of Nociception” contains 13 articles published by 73 authors from different countries united by the main focusing on the peripheral mechanisms of pain. The content covers the mechanisms of neuropathic, inflammatory, and dental pain as well as pain in migraine and diabetes, nociceptive roles of P2X3, ASIC, Piezo and TRP channels, pain control through GPCRs and pharmacological agents and non-pharmacological treatment with electroacupuncture.

Not surprisingly, the majority of papers in this collection are devoted to the functioning of TRP channels, which are the best-studied transducers of noxious stimuli but still a puzzling issue for the researchers. In the large family of TRP channels, TRPV1 and TRPA1 are best known for their leading role in key pain mechanisms. TRPV1 receptors are often co-expressed with TRPA1 in nociceptive neurons and probably can interact by means of the protein TMEM100. The details of such interactions are discussed in the well-illustrated review by Takayama and co-workers [5] summarizing the role TRP channels play in the pain states including acute pain, inflammatory pain, migraine pain, and other disorders. Much attention in this review is paid to the ANO1 protein, a calcium-activated chloride channel expressed in nociceptive neurons. The authors propose that TRP channels and ANO1 can collaborate to generate a strong pain signal in primary sensory neurons. This is an interesting “marriage” between cation and anion permeable ion channels to control the neuronal excitability. 

By focusing on TRPA1 channels, Feng and colleagues [6], using electrophysiological recordings from brainstem slices, detected activation of presynaptic polymodal TRPA1 ion channels in glutamatergic terminals synapsing on the caudal nucleus of the solitary tract neurons. The enhanced glutamatergic synaptic neurotransmission onto the second-order sensory neurons was activated by TRPA1 agonists. These findings were supported by data from the TRPA1 knockout mice where TRPA1 agonists failed to alter synaptic efficacy. The study suggests that in the caudal brainstem the input from visceral noxious stimuli can be targeted by multiple endogenous TRPA1 ligands including reactive oxygen species and probably by the classic analgesic paracetamol via its derivatives [6]. 

The original view on the role of ion channels, including TRP and other unrelated channels in nociception, was presented by Ciotu with co-workers [7]. As stated by the authors, they describe “more novel and less known features” of ion channels. In particular, they summarize not commonly considered ion channel properties such as a memory of the previous voltage and chemical stimulation, alternative ion conduction pathways, cluster formation, and role of the silent accessory subunits. This fresh view on the channels function in a realistic membrane environment should stimulate the interest to these little appreciated phenomena and can suggest new ideas for many researchers studying the pain mechanisms. 

One study in this special issue, reports the expression of TRPA1 channels not only in neurons but also in non-neuronal cells. Thus, Kameda with colleagues [8], studied the expression and activity of the pro-nociceptive TRPA1 and TRPV1 in the intervertebral disc (IVD) as its degeneration is associated with inflammatory pain. As the object of the study, they analyzed human fetal, healthy, and degenerated IVD tissues. They found that the TRPA1 agonist AITC activated inflamed IVD cells, induced expression of IL-8, but reduced disintegrin and metalloproteinase with thrombospondin motifs 5 (ADAMTS5). By comparing knockout TRPA1 versus TRPV1 mice, they further confirmed the leading role of TRPA1 in control of inflammation in IVD cells.

The experimental study by Demartini with collaborators [9], has a significant translational impact as they describe a new treatment approach for neuropathic pain. Notably, neuropathic pain is a severe disabling and often intractable condition and new therapeutic targets are of high interest. They used a drug candidate compound ADM-12 blocking the nociceptive TRPA1 receptors in the trigeminal nerve fibers. The authors used the well-established model of the neuropathic pain based on the chronic constriction injury of the infraorbital nerve. Apart from the antagonism of TRPA1 receptors, they found also other benefits of this treatment including reduced expression of TRPA1/V1 receptors and pro-nociceptive neuropeptides and cytokines, collectively contributing to diminished mechanical allodynia, a leading symptom of neuropathic pain. 

Whereas the trigeminal neuralgia is a relatively rare type of head pain, migraine headaches are very common. Migraine headache is characterized by a severe pulsatile (throbbing) type of pain also transmitted by trigeminal nerve fibers. Review by Della Pietra et al. [10] provides a new explanation for the throbbing pain in migraine by proposing a rhythmic activation, by pulsatile blood flow, of mechanosensitive Piezo channels in trigeminal fibers in meninges. Piezo ion channels (presented by Piezo1 and Piezo2 isoforms) with the highest sensitivity to mechanical stimuli were recently discovered [1]. The Piezo 1 subtype sensitive to mechanical and chemical agonists such as Yoda1, is found in trigeminal neurons along with Piezo2 subtype [11]. Della Pietra and co-authors describe the function and modulation of Piezo mechanotransducers in the trigeminovascular system as sensors generating the rhythmic migraine pain signals. The emerging field of Piezo currently attracts much attention as a new way for efficient control of Piezo-related diseases, including migraine and chronic pain. 

The review by Tardiolo et al. [12] expands our knowledge of current and novel therapeutic approaches to migraine. The particular focus in this review is on the pharmacological targets for novel drugs based on 5-HT receptor agonists (ditans), CGRP receptor antagonists (new generation of gepants), and CGRP receptor or ligand antagonists such as monoclinal antibodies (mAbs). Of note, this review contains a detailed description of these and other new emerging treatments in migraine. Further in this review, the authors present the animal models of migraine including the dural application of the inflammatory soup or high potassium to induce CSD, nitroglycerin, and transgenic migraine models. 

Apart from the clear role in migraine of the neuropeptide CGRP, another neuropeptide, substance P, often co-expressed with CGRP in nociceptive neurons, contributes to other types of pain. For decades, substance P was considered a putative promoter of pain. A review included in this collection by Chang with colleagues [13] highlights a new paradoxical role of substance P in anti-nociception, in particular, in muscle pain. The authors suggest that the anti-nociception by the substance P in muscle pain is mediated via the enhancement of the M-channel outward currents in local sensory neurons. The review contains a detailed description of ion channels modulated by substance P. Elucidating the dual role of substance P in pain control would further improve our understanding of the biological functions of this neuropeptide for better development of anti-nociceptive treatments. 

The nociceptive neurons express also a high level of P2X3 receptors activated by extracellular ATP, which is a powerful algogenic substance (Figure 1). Xiang and co-workers [14] uncovered the role of ATP-gated P2X3 receptors in analgesia by electroacupuncture in CFA-induced inflammatory pain. P2X3 receptors, opening a cationic ion channel after ATP binding, are primarily implicated in the inflammatory type of pain. The authors found that the short-term or long-term application of 100 Hz electroacupuncture increased the paw withdrawal threshold and reversed the elevation of P2X3 receptors in sensory DRG neurons. 

Mustăciosu with collaborators [15] analyzed the expression of the neuron-specific Elav-like Hu RNA-binding proteins in sensory DRG neurons in mice with the streptozotocin (STZ)-induced diabetes. These proteins presented with three isoforms HuB, HuC, and HuD, typically play a role in neurogenesis and neuronal plasticity. As the original approach, the authors compared STZ-sensitive to STZ-resistant mice with high or low glycemia, respectively. With thermal pain testing, they found that the hypoalgesia was observed only in the diabetic mice. This effect was associated with HuD downregulation and HuB upregulation, which might be related to the altered post-transcriptional control of RNAs involved in the regulation of thermal hypoalgesia. 

In their comprehensive review, Salzer and co-workers [16] discuss how the members of the big family of G-protein coupled receptors (GPCRs) control the function of different types of ion channels implicated in nociception (see also Figure 1). Notably, these CGRPs not only detect the endogenous active molecules such as opioids or cannabinoids operating as modulators of pain transducing ion channels but also represent the most important targets for various analgesic therapeutics. The authors provide in-depth analysis of GPCRs-mediated modulation of the main subtypes of ligand- (TRPs, ASIC, CaCCs), mechano- (K2P and Piezo) as well as voltage-gated (sodium, calcium, and potassium) channels implicated in nociception. 

Lee and collaborators [17] present an interesting review on the mechanisms of the dental pain, which is often extremely severe and based on the unique anatomical structure of the tooth. Along with other chemical stimuli, they paid much attention to the heat-/cold-induced nociceptive signaling via TRP type ion channels. Although thermal stimuli are the primary signals to trigger tooth pain, this cannot explain the sudden and intense tooth pain elicited by innocuous mechanical stimuli. Moreover, similar to migraine headaches, dental pain often has a pulsating character. The latter can be activated by mechanical stimulation from the rhythmic movement of dentinal fluid or deformation of tooth microstructure. Logically, as in the migraine paper by Della Pietra et al, Lee and colleagues have considered the role of the professional Piezo ion channels as detectors of these mechanical forces. As Piezo2 channels are expressed mainly in low threshold mechanoreceptive neurons, the authors proposed that, in the tooth, these neurons serve as nociceptors. Interestingly, Piezo2 were also detected in odontoblastic processes in dentinal tubules, suggesting the complex role of these mechanosensitive channels in dental pain. 

Shteinikov and co-workers [18] report the results of the experimental study on the action of the hydrophobic amines and their guanidine analogs on activation and desensitization of acid-sensing ion channels (ASIC). The ASIC3 subtype, studied here, with the highest acid sensitivity is primarily expressed in nociceptive neurons and likely implicated in various types of chronic and inflammatory pain associated with tissue acidosis. By testing a series of hydrophobic monoamines on CHO cells expressing rat ASIC3 channels, they found an interesting combination of two opposite effects of these potentially analgesic agents. This finding can explain previous contradictory results obtained with these ASIC modulators. They showed the inhibition of ASIC3 activation due to the acidic shift of proton sensitivity but they also detected the reduced desensitization of ion channels, which is normally very fast and limits the activity of these membrane proteins. 

In summary, this collection of articles provides an overview of different aspects of peripheral pain mechanisms. These papers are extending our understanding of the role of ion channels in situ, ion channel interactions, functional role of sensitization-desensitization and ion channel inactivation, endogenous modulators, and other important aspects of the functioning of excitable nociceptive neurons partnering with non-excitable cells. We believe that this issue will provide new insights into the remarkable field of pain research.

## Figures and Tables

**Figure 1:**
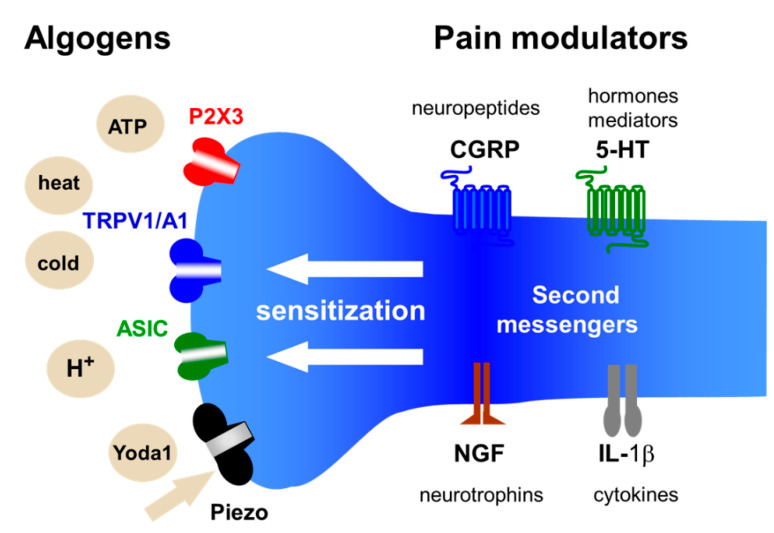
Chemical and physical stimuli (algogens) activating ion channel of nociception and pain modulators acting at the nerve terminal of the nociceptive neuron. The nerve terminal expresses specific ion channels of nociception such as ATP-gated P2X3, heat/capsaicin-sensitive TRPV1 and cold/redox-sensitive TRPA1 channels, acid-sensitive ion channels (ASICs) and mechanosensitive Piezo channels. The activity of these pain transducers can be enhanced leading to the neuronal sensitization, via metabotropic receptors, by different classes of pain modulators including neuropeptides, hormones, classical mediators, neurotrophins, cytokines, and other endogenous pro-nociceptive molecules.

## References

[1] Coste B., Mathur J., Schmidt M., Earley T.J., Ranade S., Petrus M.J., Dubin A.E., Patapoutian A. (2010). Piezo1 and Piezo2 Are Essential Components of Distinct Mechanically Activated Cation Channels. Science.

[2] Julius D., Basbaum A.I. (2001). Molecular mechanisms of nociception. Nature.

[3] Bennett D.L., Clark A., Huang J., Waxman S.G., Dib-Hajj S.D. (2019). The Role of Voltage-Gated Sodium Channels in Pain Signaling. Physiol. Rev..

[4] Pryazhnikov E., Fayuk D., Niittykoski M., Giniatullin R., Khiroug L. (2011). Unusually Strong Temperature Dependence of P2X3 Receptor Traffic to the Plasma Membrane. Front. Cell. Neurosci..

[5] Takayama Y., Derouiche S., Maruyama K., Tominaga M. (2019). Emerging Perspectives on Pain Management by Modulation of TRP Channels and ANO1. Int. J. Mol. Sci..

[6] Feng L., Uteshev V.V., Premkumar L.S. (2019). Expression and Function of Transient Receptor Potential Ankyrin 1 Ion Channels in the Caudal Nucleus of the Solitary Tract. Int. J. Mol. Sci..

[7] Ciotu C.I., Tsantoulas C., Meents J., Lampert A., McMahon S.B., Ludwig A., Fischer M.J. (2019). Noncanonical Ion Channel Behaviour in Pain. Int. J. Mol. Sci..

[8] Kameda T., Zvick J., Vuk M., Sadowska A., Tam W.K., Leung V.Y.-L., Bölcskei K., Helyes Z., Applegate L.A., Hausmann O. (2019). Expression and Activity of TRPA1 and TRPV1 in the Intervertebral Disc: Association with Inflammation and Matrix Remodeling. Int. J. Mol. Sci..

[9] DeMartini C., Greco R., Zanaboni A.M., Francesconi O., Nativi C., Tassorelli C., Deseure K. (2018). Antagonism of Transient Receptor Potential Ankyrin Type-1 Channels as a Potential Target for the Treatment of Trigeminal Neuropathic Pain: Study in an Animal Model. Int. J. Mol. Sci..

[10] Della Pietra A., Mikhailov N., Giniatullin R. (2020). The Emerging Role of Mechanosensitive Piezo Channels in Migraine Pain. Int. J. Mol. Sci..

[11] Mikhailov N., Leskinen J., Fagerlund I., Poguzhelskaya E., Giniatullina R., Gafurov O., Malm T., Karjalainen T., Gröhn O., Giniatullin R. (2019). Mechanosensitive meningeal nociception via Piezo channels: Implications for pulsatile pain in migraine?. Neuropharmacology.

[12] Tardiolo G., Bramanti P., Mazzon E. (2019). Migraine: Experimental Models and Novel Therapeutic Approaches. Int. J. Mol. Sci..

[13] Chang C.-T., Jiang B.-Y., Chen C.-C. (2019). Ion Channels Involved in Substance P-Mediated Nociception and Antinociception. Int. J. Mol. Sci..

[14] Xiang X., Wang S., Shao F., Fang J., Xu Y., Wang W., Sun H., Liu X., Du J., Fang J. (2019). Electroacupuncture Stimulation Alleviates CFA-Induced Inflammatory Pain Via Suppressing P2X3 Expression. Int. J. Mol. Sci..

[15] Mustaciosu C.C., Banciu A., Rusu C.M., Banciu D.D., Savu D., Radu M., Radu B.M. (2019). RNA-Binding Proteins HuB, HuC, and HuD are Distinctly Regulated in Dorsal Root Ganglia Neurons from STZ-Sensitive Compared to STZ-Resistant Diabetic Mice. Int. J. Mol. Sci..

[16] Salzer I., Ray S., Schicker K., Boehm S. (2019). Nociceptor Signalling through ion Channel Regulation via GPCRs. Int. J. Mol. Sci..

[17] Lee K., Lee B.-M., Park C.-K., Kim Y., Chung G. (2019). Ion Channels Involved in Tooth Pain. Int. J. Mol. Sci..

[18] Shteinikov V.Y., Potapieva N.N., E Gmiro V., Tikhonov D.B. (2019). Hydrophobic Amines and Their Guanidine Analogues Modulate Activation and Desensitization of ASIC3. Int. J. Mol. Sci..

